# Amino-acid functionalized porous silicon nanoparticles for the delivery of pDNA†

**DOI:** 10.1039/c9ra05461h

**Published:** 2019-10-07

**Authors:** Arnaud Chaix, Eduardo Cueto-Diaz, Anthony Delalande, Nikola Knezevic, Patrick Midoux, Jean-Olivier Durand, Chantal Pichon, Frederique Cunin

**Affiliations:** Institut Charles Gerhardt Montpellier, Charles Gerhardt Montpellier, Université de Montpellier UMR 5253 CNRS-ENSCM-UM2-UM1, 2 Place Eugène Bataillon 34095 Montpellier Cedex 05 France frederique.cunin@enscm.fr; Centre de Biophysique Moléculaire in Orleans (CBM) UPR4301 France; Biosense Institute, University of Novi Sad Dr Zorana Djindjica 1 21000 Novi Sad Serbia

## Abstract

Porous silicon nanoparticles as a novel platform in gene therapy, have shown to be an efficient vehicle for the delivery of nucleic acids in cells. For the first time, a family of porous silicon nanoparticles has been produced featuring an amino-acid functionalized cationic external surface aiming at pDNA complexation. The amino acid-based pDNA nanocarriers, displaying an average diameter of 295 nm, succeeded in transfection of HEK293 cells with an efficiency 300 times superior to “bare” porous silicon nanoparticles.

## Abstract 2

Scheme of gene transfer into a cell nucleus using a porous silicon nanoparticle based non viral vector.

## Graphical abstract

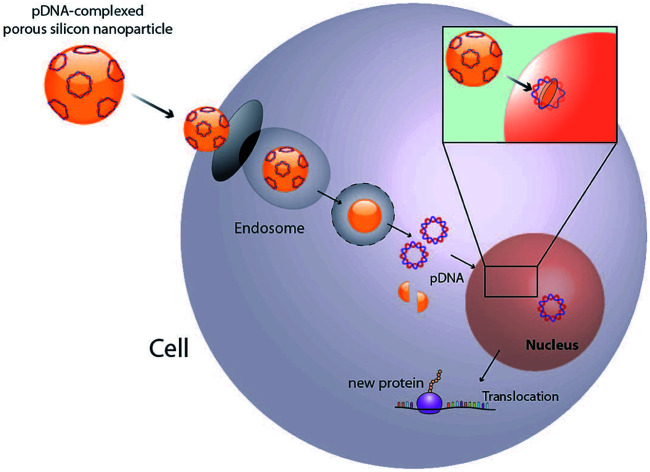

## Introduction

Gene therapy is a promising approach for the treatment of carcinogenic cells as it aims to deliver genetic materials into specific cells to produce a therapeutic effect, by either altering or correcting an abnormality or by promoting a new cell function. However, one of the main obstacles for the clinical application of this therapy aimed at malignant tumors is the lack of safe and tumor-selective gene vectors.^1^ The ideal gene carrier should provide efficient gene delivery properties, specific cell targeting, avoid inflammatory/immunogenic responses in the exposed area, and must be easily manufacturable and clinically applicable. In this regard, virus-based gene vectors, although exhibiting good transfection efficiency, yield important side effects. Multiple significant problems, such as inflammatory responses, random integration in host genome and toxicity using recombinant viral vectors have been reported.^2^ Alternative routes have been investigated leading to the development of viral-free systems. Non-viral vectors reported in the literature are synthetic chemical systems including cationic liposomes^3^ and polymers.^4^ In addition physical methods such as gene gun,^5^ electroporation,^6^ particle bombardment,^7^ and ultrasound^8^ are alternative methods for the direct injection of nucleic acids. Despite the low efficiency of non viral vectors compared to viral ones, their cost, availability, high modulability (no size limitations), with scarce generation of immunologic and no oncogenic adverse effects make them an attractive alternative for gene delivery.^9,10^

**Scheme 1:**
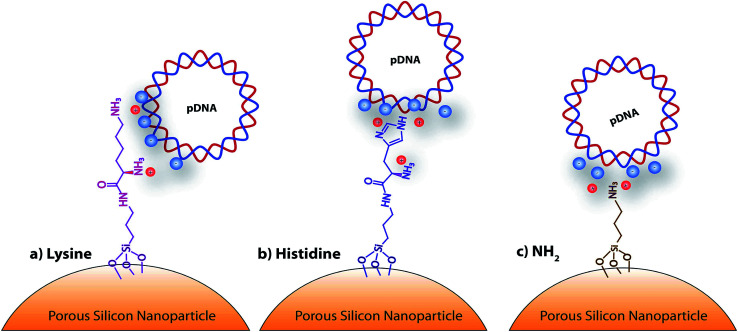
Schematic representation of the amino-acid functionalized porous silicon nanoparticles, with: (a) lysine, (b) histidine, and (c) NH_2_.

**Scheme 2:**
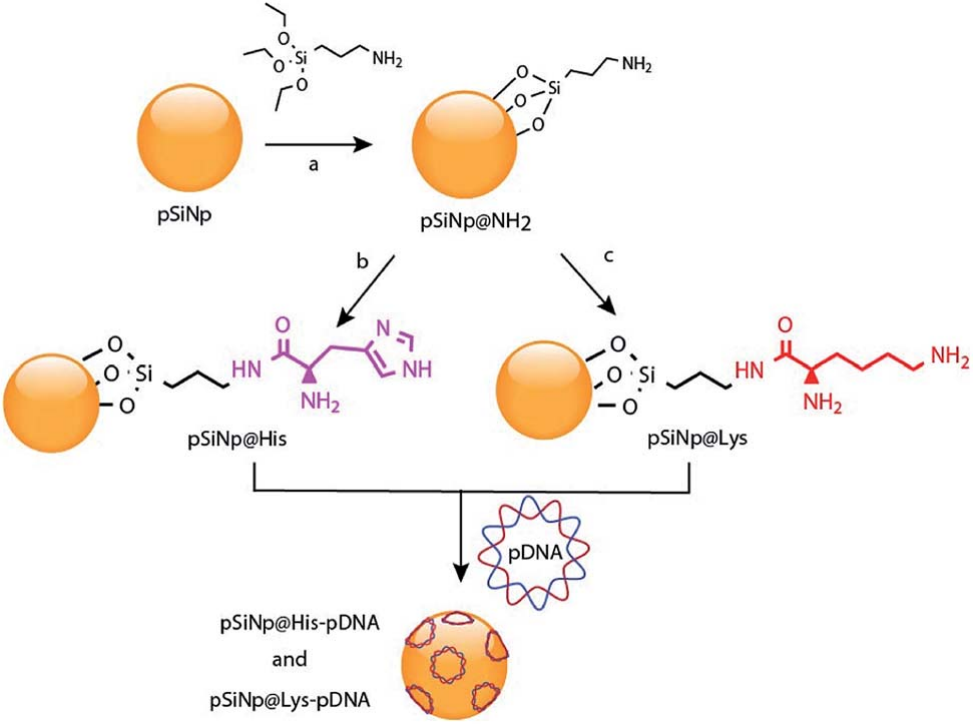
Reaction scheme for the chemical functionalization of the pSiNp with histidine and lysine, and for the complexation of pDNA: (a) APTES, toluene, 50 °C 20 h; (b) Fmoc-Hist(Boc)·OH, PyBOP, DIPEA, EtOH, rt, 18 h, CH_2_Cl_2_, TFA, 18 h; (c) Boc-Lys(Fmoc), PyBOP, DIPEA, EtOH, rt, 18 h, CH_2_Cl_2_, TFA,18 h.

Porous silicon materials and nanoparticles have proved to bear a great potential for nanomedicine applications, owing to their excellent *in vivo* biocompatibility and biodegradability.^11–16^ The major degradation by-product is silicic acid, which appears to be non-toxic to human cells.^9,10^ Herein, we report the first approach on porous silicon nanoparticles bearing positively charged amino-acids motifs (histidine, lysine) to electrostatically interact and transport the long polyanion plasmid DNA (pDNA) to cancer cells. The fusogenic feature of histidine residues at mild acidic pH, as that of endosomes, will yield the escape of the nanocomplexes from endosomes, once internalized inside cells, and favor cell transfection.^17^ Porous silicon nanoparticles (pSiNp) were prepared by anodic etching of boron doped crystalline silicon in a solution of aqueous hydrofluoric acid (HF) in ethanol following procedure previously published.^18^ Typically the pSiNp were produced after lifting-off the porous film using an electropolishing current, ultrasonication treatment, and centrifugation. The obtained pSiNp exhibited a hydrodynamic diameter of 190 nm with a polydispersity of 0.2 in accordance with transmission electron microscopy images (Fig. S1, ESI†). Three different formulations of pSiNp were prepared, two of them containing either the amino-acids lysine or histidine, and the other one bearing a primary amine in the periphery, as a control for simple positively charged surface.^19^ In this study, the pSiNp surface was first silanized with APTES followed by the peptide coupling of the amino acids (histidine and lysine) prior to pDNA complexation (Scheme 1). Silanization of pSiNp with 3-aminopropyltriethoxysilane (APTES) conducted at room temperature over 18 hours, provided the free amino functional group (pSiNp@NH_2_) allowing easy further functionalization (Scheme 2). The grafting of the amine moiety was confirmed by zeta potential measurements and DLS with an increase of the surface charge from −26.1 mV to +50.2 mV due to the presence of ammonium groups, and with an increase of the dynamic diameter from 190 nm to 295 nm respectively (Table 1, Fig. S2 and S3, ESI†). Although the DLS method is not suitable for non-spherical objects such as pSiNps, it was used in the present study in order to provide an estimation of the pSiNps size.

**Table 1:** Surface charge, size and quantification of pSiNp@amino acid in EtOH at 25 °C

Formulations	ζ-potential (mV)	μg mg^−1^pSiNp	Size (nm)
pSiNp	−26 ± 1	—	190
pSiNp@NH_2_	50 ± 1	—	295
pSiNp@His	47 ± 2	7	295
pSiNp@Lys	45 ± 5	84	295

The dramatic increase in the hydrodynamic diameter of the intermediate formulation pSiNp@NH_2_ was ascribed to solvation provoked by the presence of the amine groups (Table 1). In addition APTES can polymerize and form a multilayer at the surface of the nanoparticles thus increase their hydrodynamic diameter. Finally, this increase of the hydrodynamic diameter could also result from the fact that porous silicon nanoparticles are neither spherical nor isotropic 3D nanoparticles. Attenuated total reflectance Fourier-transform infrared (ATR-FTIR) spectroscopy, as presented in Fig. S4, ESI,† also confirmed anchoring of the aminated function. The quantification of the attached APTES moiety was determined by elemental analysis (2.0% (N), 10.4% (C)) with a grafted amount of 1.16 mmol (APTES) per gram of pSiNp (Fig. S5, ESI†). After silanization, the two protected amino acid motifs were condense through peptide coupling. Benzotriazol-1-yl-oxytripyrrolidinophosphonium hexafluorophosphate (PyBOP) was the selected coupling reagent as it promotes rapid binding and yields less noxious by-products; *N*,*N*-diisopropylethylamine (DIPEA) was chosen as the organic base.

Next, Boc and Fmoc were successively removed by means of TFA and piperidine addition, leading to the final formulations, pSiNp@Lys and pSiNp@His. The supernatant in both cases was collected for dosing the histidine and lysine and then for quantification of the histidine and lysine immobilized on the pSiNp. The reaction progress was monitored by zeta potential measurement, DLS and FTIR spectroscopy. Zeta potential measurement of pSiNp@Lys and pSiNp@His was carried out after sonication of the samples for 90 s to minimize aggregation. The resulting values at +45 mV and +47 mV respectively indicated successful immobilization and deprotection of the amino acids at the surface of the nanoparticles (Table 1 and Fig. S2, ESI†). The hydrodynamic diameter of both formulations resulted in a similar value of 295 nm (Table 1 and Fig. S3, ESI†). The FTIR spectra revealed two characteristic bands at 790 cm^−1^ and 800 cm^−1^ attributed to the bending vibrations of primary and secondary N–H bonds present in the three formulations, but absent in the pSiNp, confirming the successful functionalization of the pSiNp (Fig. S4, ESI†). In addition, the presence of intense narrow bands at 1050 cm^−1^ was ascribed to *ν*_s_ (Si–O–Si) confirming the APTES grafting into the silicon nanoparticles. Deprotection and Fmoc removal were then conducted in a two steps sequence. First, the removal of the acidic proton at the 9-position of the fluorene ring system by a mild base, (secondary cyclic amine (piperidine)) was performed, and secondly the subsequent β-elimination was conducted yielding a highly reactive species such as dibenzofulvene, and intermediate that were immediately trapped by the organic base forming the stable adduct (for details, see ESI†). Fmoc-aminoacid assay performed by means of UV-Vis spectroscopic experiments at 290 nm, allowed the quantification of each attached amino acid, thus indicating the grafting of 0.045 mmol (histidine) and 0.575 mmol (lysine) per mg of pSiNp (more details in Fig. S5 and S6, ESI†). The different grafting efficiency was ascribed to the high steric hindrance and low solubility of the histidine moiety.

**Fig. 1:**
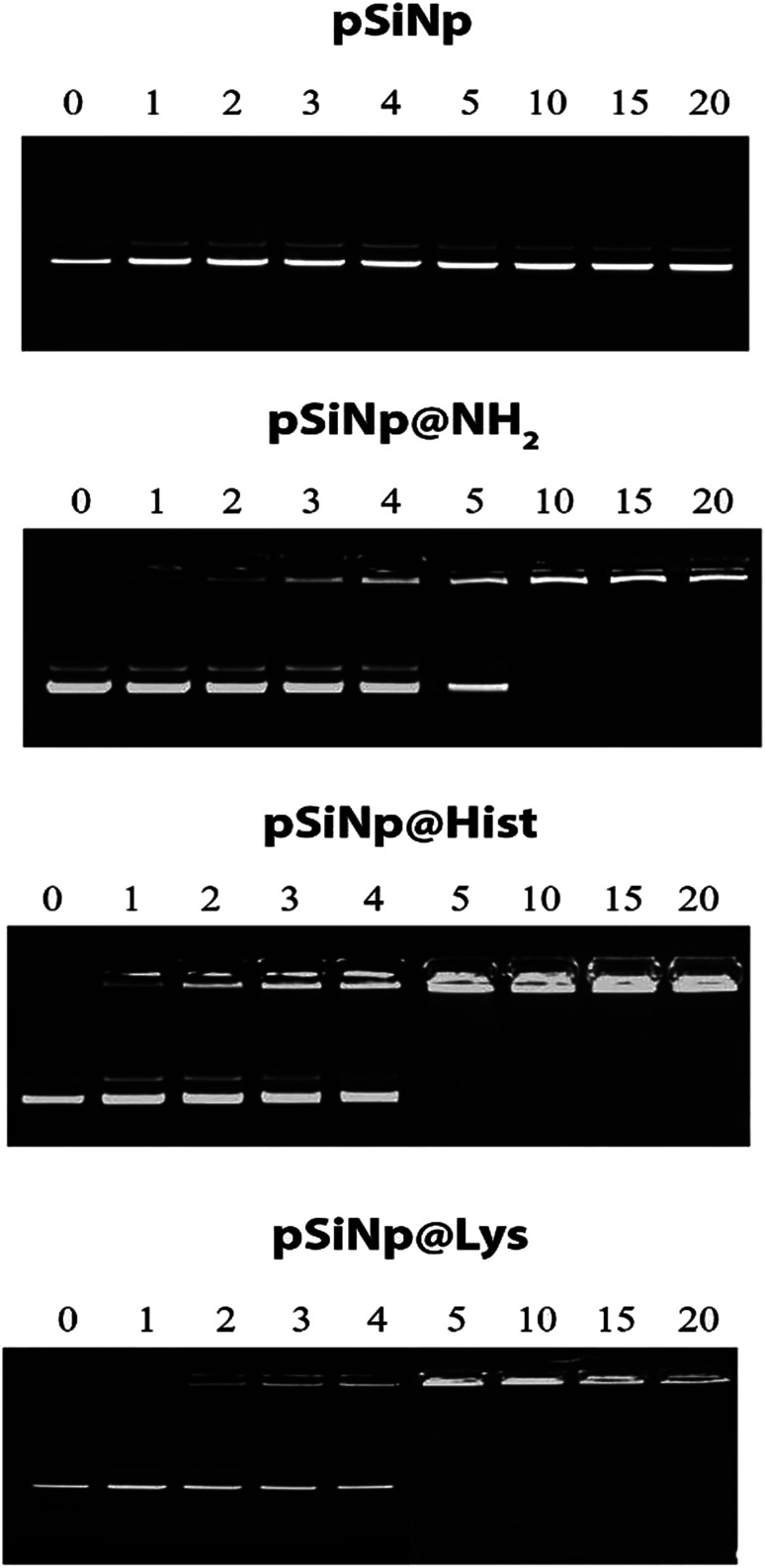
Gel retardation assay demonstrating DNA@nanoparticle complexation. A constant amount of pDNA (1 μg) was complexed with nanoparticles at nine different ratios in HEPES (10 mM, pH 7.4).

The complexation efficiency of plasmid DNA was monitored at physiological pH (7.4) for the three formulations pSiNp@NH_2_, pSiNp@Lys and pSiNp@His by gel shift experiments (Fig. 1). No complexation was observed between the non-functionalized pSiNp and pDNA whatever the ratio used. As pSiNp exhibits a surface charge of – 26 mV due to the presence of silanols at their surface, they did not show affinity for negatively charged pDNA. In contrast pSiNp@NH_2_, pSiNp@Lys and pSiNp@His with surface charge of +50 mV, +45 mV, +47 mV respectively, efficiently complexed pDNA. For most of SiNp derivatives, the complexation started to form at 1/1 and then full complexation was observed at a ratio of 1/5 (Fig. 1).

**Fig. 2:**
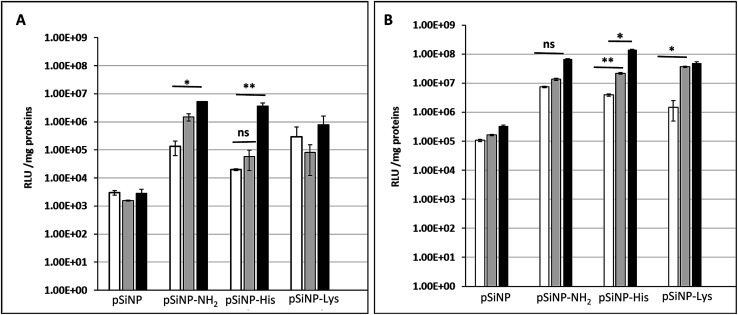
Transfection efficiency: luciferase activities (RLU per mg protein) measured 48 h following transfection of HEK 293 human cells with indicated silicon nanoparticles formulated with pDNA encoding luciferase gene at a ratios of 1/1, 1/5 and 1/10 (white bar, grey bar and black bar, respectively) in absence (A) or presence of 100 μM chloroquine (B).

The transfection efficiency of all formulations pSiNp@NH_2_, pSiNp@Lys and pSiNp@His at 1/1, 1/5 and 1/10 ratios was investigated on HEK 293 human cells using pDNA encoding luciferase reporter gene expression as a read out (Fig. 2). Except for pSiNp, all formulations did result into efficient gene transfer. The level of gene expression is dependent on the ratio for pSiNp@NH_2_ and pSiNp@His. For pSiNp@Lys formulation, there is no clear significant difference of luciferase units whatever the ratio used. For the others, the transfection efficiency was higher at a ratio of 1/10, the values were close to 10^7^ RLU per mg proteins (Fig. 2A). We assessed the effect of chloroquine, known as endosomolytic agent reported to improve drastically the cell transfection.^20,21^ The transfection efficiency was highly improved at least by 10-fold as seen for pSiNp and by 100-fold for pSiNp@NH_2_, pSiNp@His and pSiNp@Lys compared to transfection made in absence of chloroquine (Fig. 2B). These data inform that the endosomal escape was not so efficient even when pSiNp was substituted with histidine, known to induce efficient endosomal escape *via* proton sponge like effect. This behaviour is quite different to that observed for cationic polymers or liposomes, which become more efficient once substituted with histidine residues.^22^ Note that this improvement could be also due to another reported effect of chloroquine on the dissociation of pDNA complexes.^23^ Indeed, too tight complexation of DNA with the vector could hamper the recognition by the transcription machinery reducing the gene expression.

**Fig. 3:**
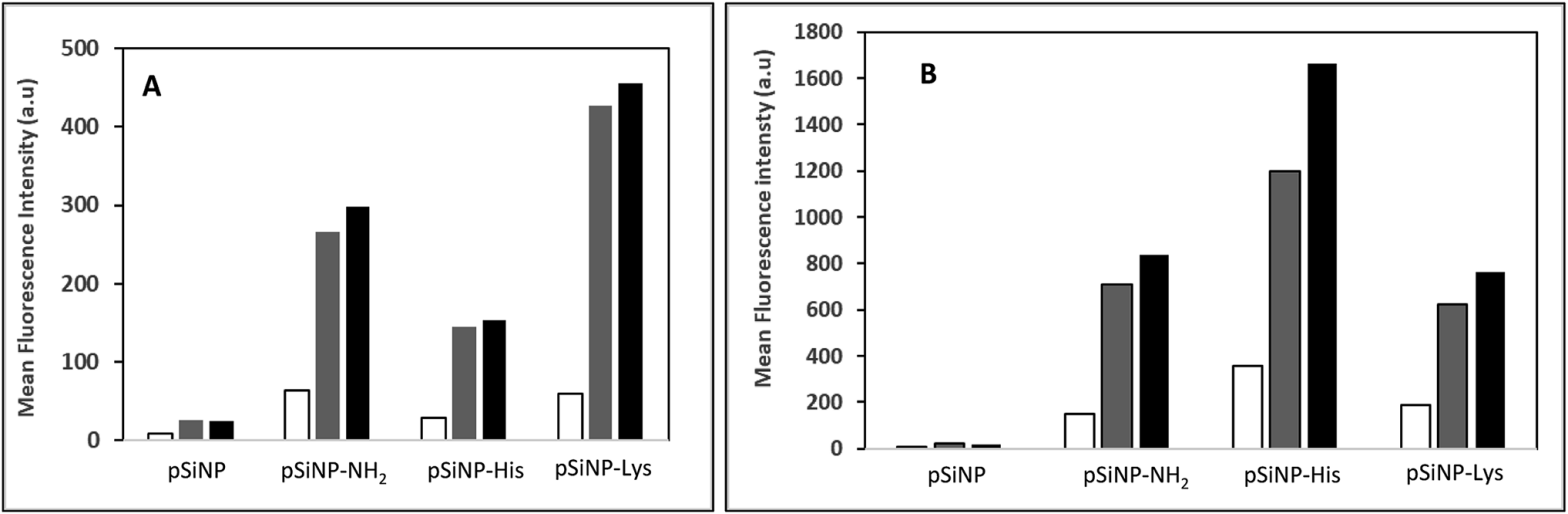
Cell uptake efficiency: cells were incubated for 2 h at 37 °C with indicated silicon nanoparticle formulated with 1 μg of fluorescein-labelled pDNA either at ratio 1/3 (A) or 1/10 (B). Cell associated fluorescence intensity was recorded before and after quenching with Trypan blue to get the surface associated (white bar) and intracellular-associated (grey bar) cell fluorescence intensities. Black bars correspond to cell fluorescence intensities recorded upon monensin treatment. Values are mean of cell fluorescence intensities recorded by flow cytometry with 10 000 events.

The cell uptake efficiency was evaluated by flow cytometry using formulations made with fluorescein-labelled pDNA (Fig. 3). The mean fluorescence intensity (MFI) measured is related to the amount of pDNA. The surface and intracellular associated fluorescence intensities (Fig. 3, white and grey bars, respectively) were evaluated thanks to the well-known quenching of fluorescein label with Trypan blue. PSiNp formulation was hardly taken up by cells compared to other formulations, which explains the low transfection efficiency. In contrast, the intracellular associated fluorescence intensities were at least 3-fold more than the surface associated one for pSiNp@NH_2_, pSiNp@His and pSiNp@Lys revealing that they were efficiently uptaken. The uptake was formulation and ratio-dependent. At low ratio (1/3), the uptake capacity of different formulations could be ranked as follows: pSiNp@Lys > pSiNp@NH_2_ > pSiNp@His > pSiNp. When made at a higher ratio (1/10), the order was different: pSiNp@His > pSiNp@Lys = pSiNp@NH_2_ > pSiNp. To assess if internalized DNA formulations were present in acidic compartment, cells were treated with monensin in presence of a neutral phosphate buffer (PBS). As being an ionophore, monensin treatment can reverse the acidity of the milieu. Since the fluorescein fluorescence intensity is quenched in acidic medium, it can be restored at neutral pH. Data reveal that at low ratio, all complexes were not localized acidic compartments because no significant differences of the MFI before and after monensin treatment (Fig. 3, grey *versus* black bars). At ratio 1/10, similar observation can be made except for pSiNp@His, for which the MFI increased a bit though not significantly. Last, cellular viability was also measured by XTT according to the manufacturer's instructions. In the absence of chloroquine, transfection made with all formulations used at ratio of 1/1 did not induce any cytotoxicity. At higher ratios, a low cytotoxicity (20%) was observed except for pSiNp@His formulation, which did not result in any reduction of cell viability (Fig. S7, ESI†). The substitution of porous silicon with histidine residues results in a preservation of cell viability as we observed for the drastic reduction of polyethyleneimine cytotoxicity once it was substituted.^24^ In cells treated with chloroquine, the toxicity (up to 40%) was higher likely due to the effect of this treatment. Again, there is a distinction of pSiNp@His formulation, which did not lead to any viability alteration. By contrast, a substantial cytotoxicity was measured (∼40%) in the presence of chloroquine for pSiNp@NH_2_ and pSiNp@Lys used at ratios of 1/3 and 1/10. This is in line with our previous conclusions.

Porous silicon nanoparticles were chemically modified bearing an amino acid functionalized cationic surface for pDNA complexation and cell transfection *in vitro*. The formulations of histidine-functionalized pSiNp gave very promising results for plasmid delivery. Transfection efficiency and uptake data indicate that, as any delivery vehicle, a higher ratio is required to complex and protect pDNA. We demonstrate that the amino-acid functionalized nanoparticles succeed in transfection of HEK cells, and that they are non-toxic to the cells 48 hours after transfection. In this work, a new type of material for transfection and for plasmid delivery was explored. pSiNps did not generate adverse effects and showed limited toxicity compared to usual cationic vectors made of polymers or liposomes.

## Conflicts of interest

There are no conflicts to declare.

## Supplementary Material

RA-009-C9RA05461H-s001
