# Supplementary material for: Amino-acid functionalized porous silicon nanoparticles for the delivery of pDNA
Source: RSC Adv. 2019 Oct 7;9(55):31895–9. doi: 10.1039/c9ra05461h (PMC9072902; doi:10.1039/c9ra05461h)
Supplement: RA-009-C9RA05461H-s001 [file RA-009-C9RA05461H-s001.pdf]

Electronic Supplementary Material (ESI) for RSC Advances.
This journal is © The Royal Society of Chemistry 2019

SUPPLEMENTARY INFORMATION

Amino-acid functionalized porous silicon nanoparticles for the delivery of pDNA

Arnaud Chaix, Eduardo Cueto-Diaz, Anthony Delalande, Nikola Knezevic, Patrick Midoux, Jean-Olivier Durand, Chantal Pichon, Frederique Cunin

Experimental section

I. Preparation of the pSiNP

I.1. Synthesis of the pSiNP

Boron-doped p^{++}-type Si (0.8-1.2 mΩ.cm resistivity, <100> orientation) from Siltronix (France) was electrochemically etched in a 3:1 (v:v) solution of aqueous 48% hydrofluoric acid (HF):absolute ethanol (Sigma-Aldrich). Etching was performed in a Teflon cell with a platinum ring counter electrode. A constant current of 200 $mA.cm^{-2}$ was applied for 150 s, and then the sample was rinsed 3 times with ethanol. The porous layer was then removed from the substrate by application of a constant current of 4 $mA.cm^{-2}$ for 250 s in an electrolyte solution containing 1:20 (v:v) aqueous 48% hydrofluoric acid:absolute ethanol. After 3 rinses with ethanol, the porous layer was placed in ethanol in a glass vial. After degassing the sample for 20 min under a nitrogen stream, the porous silicon film was fractured by ultrasonication during 16 h. The largest particles were then removed by spinning them down by centrifugation at 3,000 rpm for 2 min (Minizine, Eppendorf). In order to remove the smallest particles, the solution was finally centrifuged at 14,000 rpm for 30 min (centrifuge Eppendorf 5804). The pellet was then re-dispersed in absolute ethanol.

I.2. Characterizations of the pSiNP

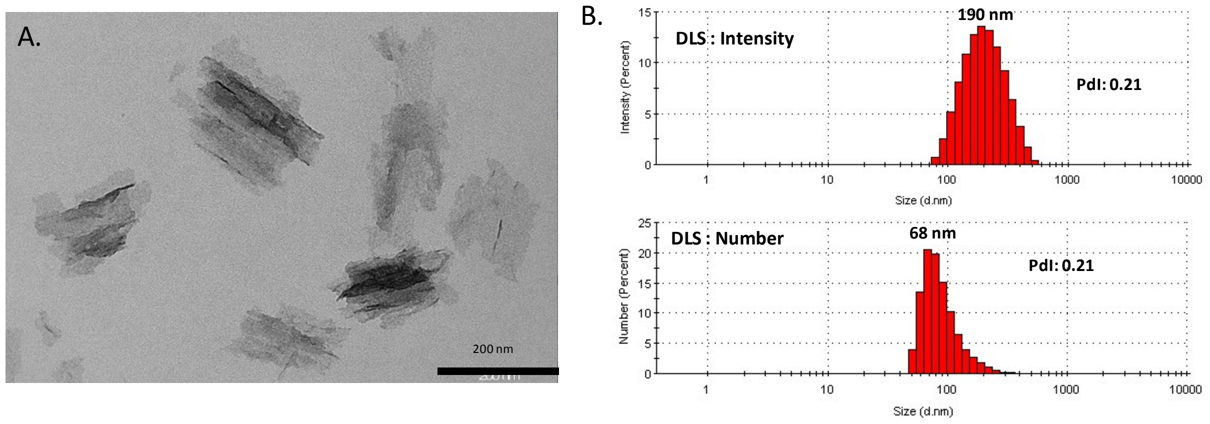

Figure S1. Characterizations of pSiNp. A) Transmission electron microscopy (TEM), B) Particle size distribution in number and intensity, measured by Dynamic Light Scattering.

II. Functionalization of the pSiNP

II. 1. Synthesis of the pSiNp@NH_2

120 mg of pSiNp were suspended in 15 mL of anhydrous toluene anhydrous and 200 µL of (3-aminopropyl) triethoxysilane (APTES) was added. The colloidal suspension was then kept

under stirring at 50°C for 20 hours. After that, the pSiNp were centrifuged for 15 minutes at 14,000 rpm and rinsed 5 times with absolute ethanol leading to the formulation, **pSiNp@NH$_2$**. Elemental analysis: C 10.4 %, H 2.3 %, N 2.0 %. Quantification: 1.16 mmol(APTES)/g (pSiNP).

II. 2. Synthesis of pSiNp@His

20 mg of pSiNp@NH$_2$ were redisperded in 2 mL of absolute ethanol, after that *N*□-(((9*H*-fluoren-9-yl)methoxy)carbonyl)*N*□-(*tert*-butoxycarbonyl)histidine(Fmoc-His(Boc)-OH-CHA) (37 mg, 63 µmol, 1.1 eq), (Benzotriazol-1-yloxy) tripyrrolidinophosphonium hexafluorophosp-hate (PyBOP) (35 mg, 67 µmol, 1.1 eq) and *N*, *N*-diisopropylethylamine (DIPEA) (11 µl, 65 µmol, 1.1 eq) were consecutively added. The reaction was kept under stirring for 18 hours at room temperature. Afterwards, the nanoparticles were centrifuged and rinsed with absolute ethanol and treated with a solution of TFA/DCM (2mL, 1:1) for 3 minutes at room temperature. The nanoparticles were then, rinsed three times with absolute ethanol and Fmoc deprotection was conducted by adding a solution of pyperidin/DMF (2 mL, 1:1), yielding **pSiNp@His**. In a further step, the colloidal suspension was centrifuged at 14,000 rpm for 15 minutes and the supernatant was collected for spectrophotometric Fmoc-quantitation assay, thus leading to an amount of 45 µmol of histidine grafted per gram of nanoparticle.

II. 3. Synthesis of pSiNp@Lys

20 mg of pSiNp@NH$_2$ were redisperded in 2 mL of absolute ethanol, after that N^{α}-(((9H-fluoren-9-yl)methoxy)carbonyl)-Nt-(tert-butoxycarbo nyl)histidine (Fmoc-Lys(Boc)-OH) (30 mg, 63 µmol, 1.1 eq), (Benzotriazol-1-yloxy) tripyrrolidinophosphonium hexafluorophosp-hate (PyBOP) (35 mg, 67 µmol, 1.1 eq) and *N*, *N*-diisopropylethylamine (DIPEA) (11 µl, 65 µmol, 1.1 eq) were consecutively added. The reaction was kept under stirring for 18 hours at room temperature. Afterwards, the nanoparticles were centrifuged and rinsed with EtOH abs. and treated with a solution of TFA/DCM (2mL, 1:1) for 3 minutes at room temperature. The nanoparticles were then, rinsed three times with absolute ethanol. and Fmoc deprotection was conducted by adding a solution of pyperidin/DMF (2 mL, 1:1), yielding **pSiNp@Lys**. In a step further, the colloidal suspension was centrifuged at 14,000 rpm for 15 minutes and the supernatant was collected for spectrophotometric Fmoc-quantitation assay. Thus, leading to an amount of 0,575 mmol of Lysine grafted per gram of nanoparticle.

III. Characterizations of the functionalized nanoparticles

Particle sizes and size distributions were determined in ethanol by dynamic light scattering (DLS) using a Malvern Zetaziser particle sizer 380 (PSS) at a fixed angle of 90°. Infrared spectra were recorded on Nicolet IS5 spectrometer with the ATR ID5 module. UV-vis absorption measurements were performed using a lambda 35 Perkin Elmer spectrometer. Transmission electron microscopy (TEM) images were obtained on JEOL 1200 EXII instrument.

III. 1. Zeta potential

Two drops of diluted functionalized porous silicon nanoparticles in absolute ethanol were added to 1 mL of absolute ethanol. The zeta measurements of the functionalized pSiNP were performed on Malvern Nanozetasizer.

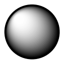

pSiNP

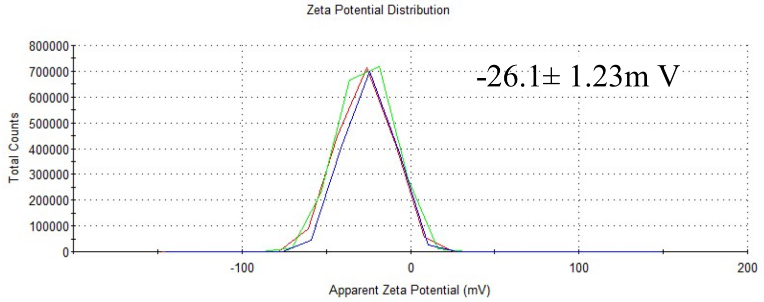

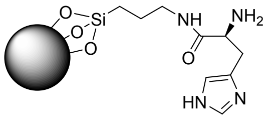

pSiNP@Hist

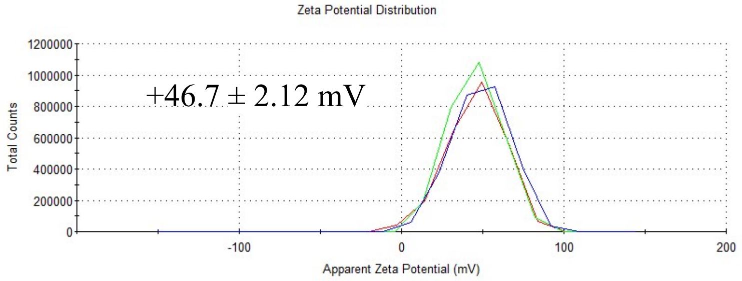

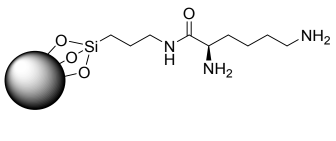

pSiNP@Lys

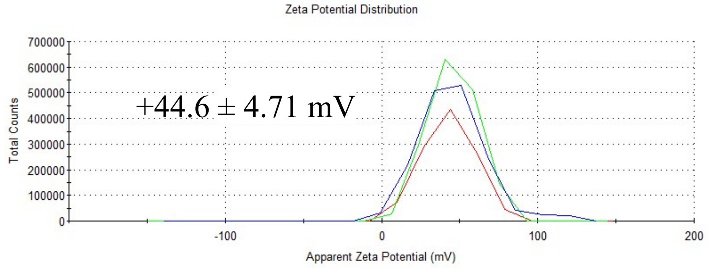

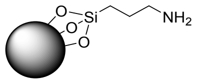

pSiNP@NH_2

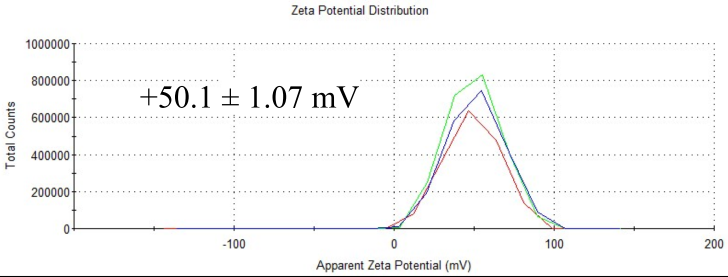

Figure S2. Values recorded for zeta potential on the different nanostructures in ethanol. The negative charge value for pSiNP is due to the partial oxidation of the nanoparticles with the presence of silanol species at the surface of the particles. After the aminoacid grafting the charge of the systems switches to positive values whereas the pSiNP remains negative. This can be ascribed to the absence of amino-terminated groups beared by the lysine and histidine motifs.

III. 2. Dynamic light scattering

The DLS measurements of the materials were performed on a Malvern nanozetasizer in a diluted ethanol solution.

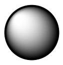

pSiNP

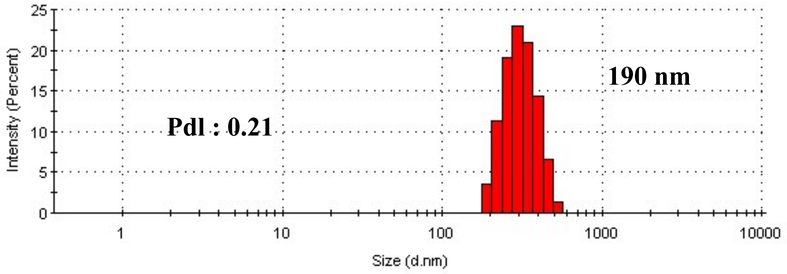

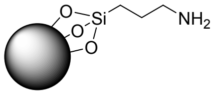

pSiNp@NH_2

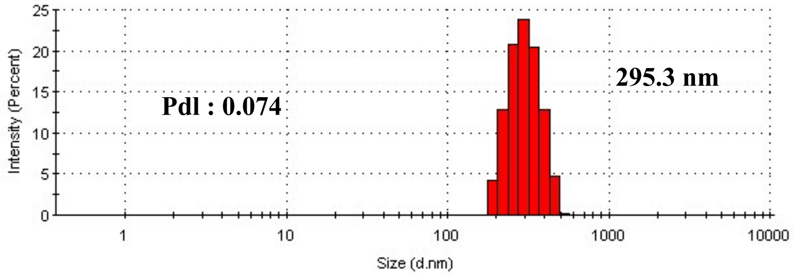

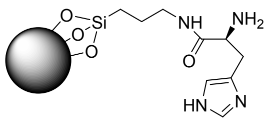

pSiNP@Hist

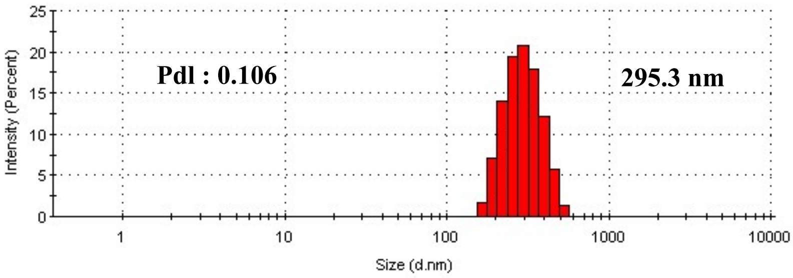

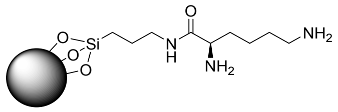 pSiNP@Lys	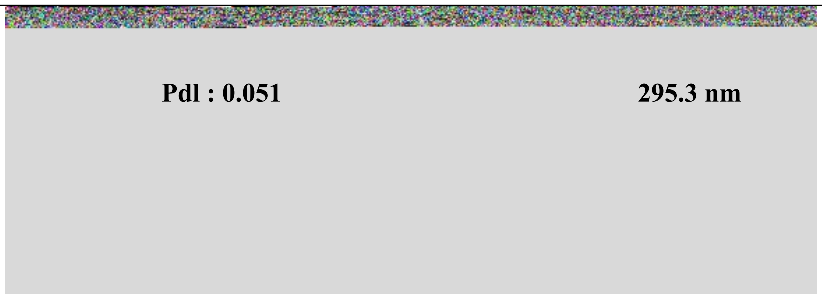

Figure S3. Size distribution by intensity of pSiNP and functionalized pSiNP.

III. 3. Infrared spectroscopy:

Infrared spectra were recorded on a Nicolet IS5 spectrometer with the ATR ID5 module. The narrow band centered at ≈800 cm^{-1} can be ascribed to the bending of NH/NH_2[1], which is compatible with the presence of APTES (red) or aminoacid moieties (blue). On the other hand, at ≈2100 cm^{-1} is located the Si-H stretching band, which which is observed to decrease upon surface oxidation (vibronic Si-O-Si and Si-O (≈1150 cm^{-1})[2] and functionalization.

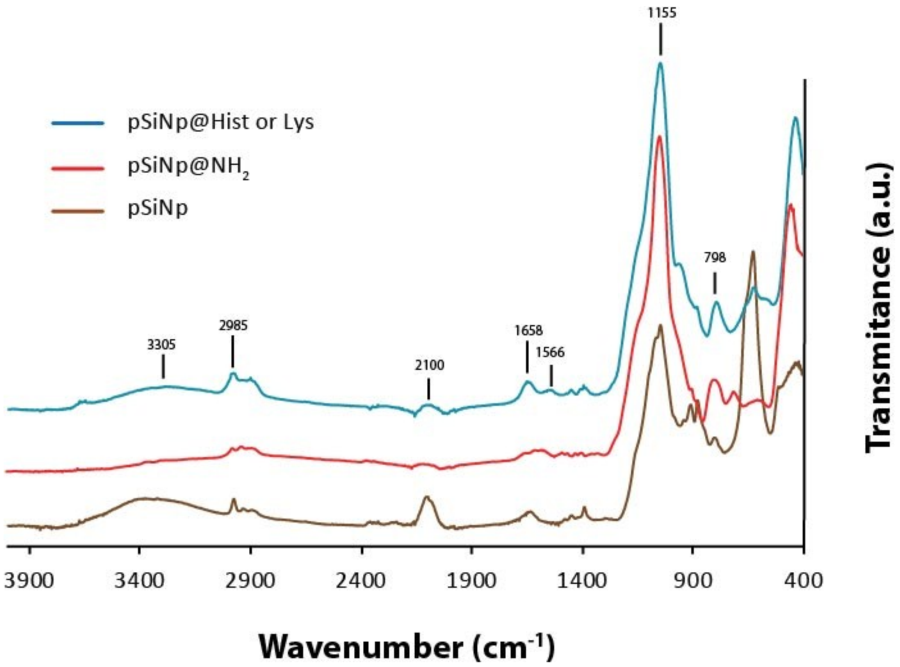

Figure S4. DRIFTS spectra of the synthesized materials. **pSiNp** (brown), **pSiNp@NH$_2$** (red) and both **pSiNp@Lys** and **pSiNp@His** (Blue).

III. 4. Quantification of the amino acids

Dosing of amino acids: the Fmoc group was removed by sonication for 30 min of pSiNP in DMF (1 mL) and piperidine (1 ml). After centrifugation for 15 min at 14 000 rpm, the supernatant was collected and used for the determination by UV-vis spectroscopy (figure S5). The spectrometric measurements were performed at the absorption wavelength of 290 nm of

the dibenzofulvene–piperidine adduct,[3] which corresponds to a molar extinction coefficient of 5253 $mol^{-1}\cdot cm^{-1}\cdot L$. The recorded absorption value and the volume were entered into the Lambert–Beer's equation, together with the substance-specific molar absorption coefficient at 290 nm. Then the number of moles of grafted aminoacids was obtained by knowing the amount of dibenzofulvene–piperidine adduct present in the supernatant.

$A = \varepsilon c l$

A = 0.86, $\varepsilon = 5.3 \times 10^{3}$ $mol^{-1}\cdot cm^{-1}\cdot L$ => $c = 1.6 \times 10^{-4}$ M of aminoacid grafted

V= 2 mL => $n = 3.2 \times 10^{-7}$ mol => **m= 84.3 μg of Lysine/mg pSiNp**

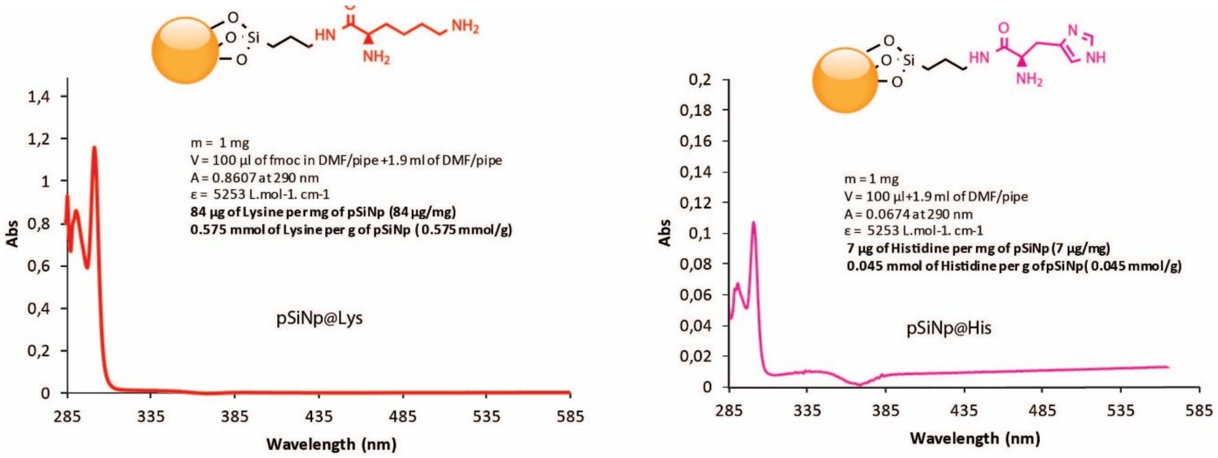

Figure S5. UV-vis spectrum of Fmoc group.

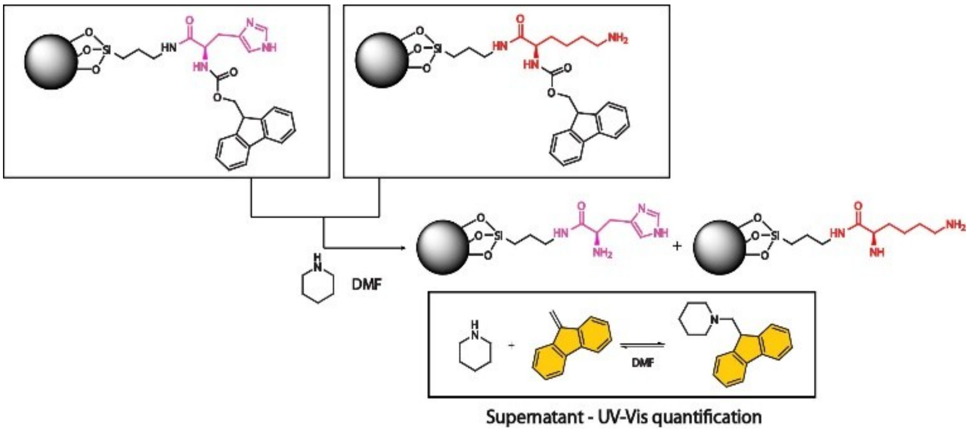

Figure S6. Schematic illustration of the Fmoc deprotection

IV. Biological studies with pDNA

4.1. pDNA studies

Plasmid DNA

pCMV-luc (pTG11033, 9514 bp, Transgene S.A., Strasbourg, France) was a plasmid DNA (pDNA) encoding the firefly luciferase (luc) gene under the control of the human cytomegalovirus (CMV) prometer. pNFCMV-luc was a homemade pDNA of 7.5 kb encoding the firefly luciferase gene under control of the strong cytomegalovirus (CMV) promoter. Five consecutive kB motifs (termed NF) that recognize the NFkB transcription factor have been inserted upstream of the prometer of pNFCMV-luc. Super coiled plasmid DNA (pDNA) was isolated from bacteria by standard alkaline lysis method, and purification was carried out with the QIAGEN Mega Kit (QIAGEN, Courtaboeuf, France).

pDNA/pSiNP

PSiNP, pSiNP-NH_2, pSiNP-His, pSiNP-Lys at 8 mg/mL, 15 mg/mL, 5 mg/mL and 5.4 mg/mL in ethanol, respectively, were sonicated for 30 min before use. pDNA/pSiNP formulations (50 µl final volume) were prepared at different pDNA/pSiNP weight ratio (µg:µg) from 1/3 to 1/10. pSiNP were added to 150 mM NaCl aqueous solution containing pDNA, and the mixture was kept for 30 min at room temperature before transfection. pDNA/pSiNP formulations were freshly prepared before use.

Gel shift assay

Complexes formation between pSiNP and pDNA was evaluated by the gel-shift assay. Each pDNA/pSiNP dispersion with desired weight ratio was mixed with 6µl loading buffer bromophenol blue/ xylene cyanol) and then loaded on a 3% (W/V) agarose gel containing SYBR green dye. The amount of pDNA loaded into each well was 0.4 µg in a total volume of 10 µL. The electrophoresis was performed under 100V for 45 min. pDNA bands were visualized under UV irradiation at 304 nm (Gene Flash System transilluminator, Syngene mounted with Pulnix TM-300 camera).

The figure presents the combination of two pictures of two separate gels done at several nanoparticle concentrations explaining why backgroung appears different.

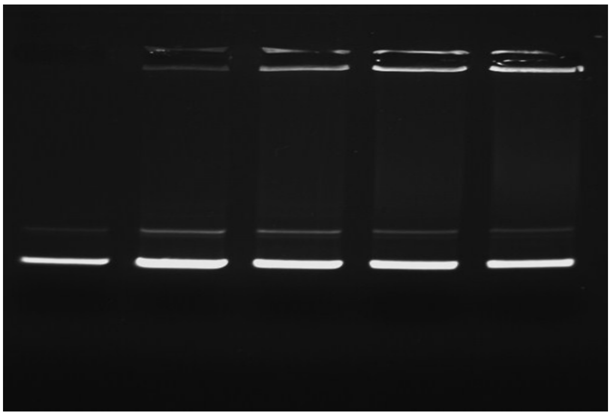

Gel 1 – pSiNp@Lys (control; 1; 2; 3; 4)

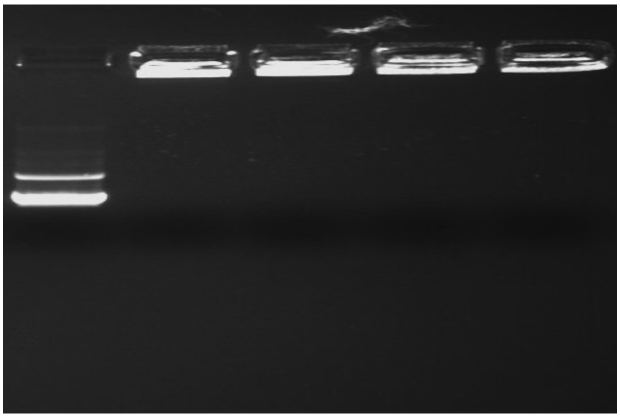

Gel 2 – pSiNp@Lys (control; 5; 10; 15; 20)

In vitro transfection experiments

Two days before transfection cells were seeded in 24 well culture plates density of 10^5 cells/cm². At the time of the transfection, the cells were 80 % confluent. Cells were then incubated for 4h at 37 °C with 0.5 mL (2.5 µg pCMV-luc) of the transfecting solution. Then, the medium was removed, replaced by fresh complete culture medium and the cells were cultured for 2 days before measurement of the luciferase activity.

Luciferase activity

Luciferase activity was measured using a lumat LB9507 luminometer (Berhold, Wildbach, Germany) according to Midoux and Mosigny[4]. The amount of proteins was determined by a modified bicinchoninic assay and the luciferase activity (relative light units, RLU) was normalized to RLU per miligram of extracted proteins.

Cell viability

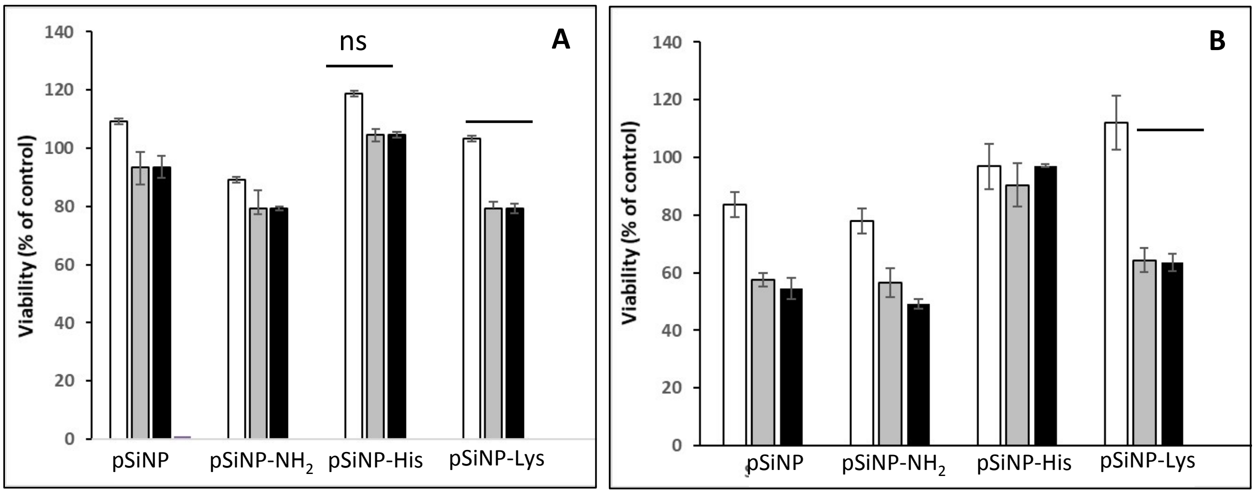

Figure S7. Cell viability of pSiNp, pSiNp@NH_2, pSiNp@Lys and pSiNp@His formulated with pDNA at ratio 1/1 (White), 1/3 (Grey) and 1/10 (Black) during 4h at 37°C in the absence (A) or presence of chloroquine (B) after 48h transfection.

References

1 Pretsch, E.; Bühlmann, P.; Badertscher, M. *Structure. Determination of Organic Compounds; Springer: Berlin, Germany*, 2009; DOI: 10.1007/978-3-540-93810-1.
2 Kaukonen, A. M., Laitinen, L., Salonen, J., Tuura, J., Heikkila, T., Limnell, T., Hirvonen, J., Lehto, V. P., Eur. J. Pharm. Biopharma., 2007, **66**, 348-356.
3 Eissler, S., Kley, M., Bächle, D., Loidl, G., Meier, T., Samson, D. *J. Pept. Sci*., 2017, **23**, 757.
4 Midoux P., Monsigny M. *Bioconjug Chem.,* 1999, **10**(3), 406-11.
